# Congenital pyriform sinus fistula presenting as a neck abscess in a newborn: A case report

**DOI:** 10.1097/MD.0000000000017784

**Published:** 2019-11-01

**Authors:** Yishu Teng, Shuling Huang, Guowei Chen, Zhixiong Xian, Saihong Han, Lan Li

**Affiliations:** Department of Otolaryngology, Shenzhen Children's Hospital, Shenzhen, Guangdong, China.

**Keywords:** CO_2_ laser, congenital pyriform sinus fistula, suspension laryngoscopy

## Abstract

**Rationale:**

Congenital pyriform sinus fistula (CPSF) is a branchial abnormality originating from the third or fourth branchial pouch and is an important cause of anterior cervical abscess in children. Here we present a case of neck abscess in a newborn that was diagnosed as CPSF.

**Patient concerns:**

A male infant with a birth weight of 3660 g was admitted to hospital 25 minutes after birth after discovery of a cystic mass with extensive skin swelling in the left side of the neck. B-mode ultrasonography of the left neck showed an anterior cervical cystic mass of indeterminate nature.

**Diagnosis:**

Congenital pyriform sinus fistula.

**Interventions:**

The neck abscess was incised and drained under general anesthesia. Examination under suspension laryngoscopy revealed a pyriform sinus fistula. Laser cauterization was performed simultaneously. The wound was dressed and anti-inflammatory treatment was provided.

**Outcomes:**

The neck wound healed uneventfully. After 3 months, the fistula was confirmed to be closed by laryngoscopy under general anesthesia. No recurrence was detected during 9 months of follow-up.

**Lessons:**

CPSF should be strongly suspected in a patient with an unexplained neck abscess or recurrent acute suppurative thyroiditis, especially on the left side.

## 1 Introduction

Patients with congenital pyriform sinus fistula (CPSF) generally present with recurrent neck swelling, pain, and/or a fistulous orifice with purulent discharge in the anterior or lateral neck region. CPSFs tend to be misdiagnosed as acute suppurative thyroiditis, neck cellulitis, neck abscess, or another disorder, such as a thyroglossal cyst or second branchial cleft.^[1]^ A relatively rare clinical manifestation in neonates is a non-infectious, compressible neck mass.^[2]^ The possibility of this disease should be kept in mind in a patient with an acute suppurative thyroid inflammatory response, especially on the left side and with repeated episodes, and timely examination is needed for a definitive diagnosis.^[3,4]^ Several imaging investigations, including barium swallow X-ray, computed tomography (CT), magnetic resonance imaging (MRI), suspension laryngoscopy, and esophagoscopy, are generally used to confirm the diagnosis.^[5]^

## 2 Case report

**Figure 1:**
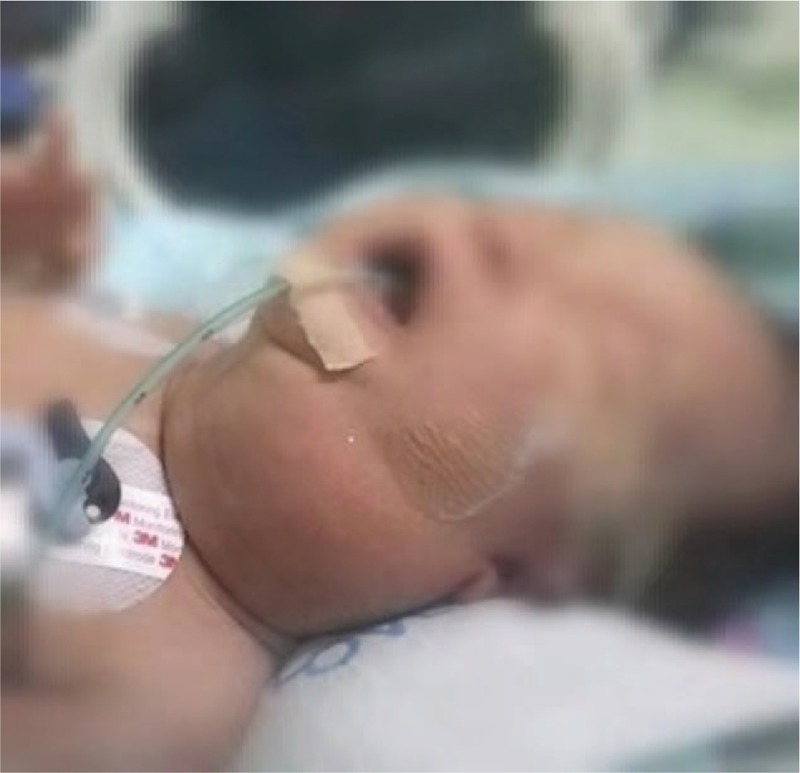
Preoperative photograph of a child with an obvious left-sided neck abscess.

A male neonate was admitted to our Department of Pediatrics directly from the delivery room 25 minutes after birth following discovery of a cystic mass with extensive skin swelling in the left side of the neck (Fig. 1). He had a birth weight of 3660 g and 1-minute and 5-minute Apgar scores of 10. The mother was gravida 2, para 1, and gave birth at 40 weeks’ and 3 days’ gestation. Her amniotic fluid was clear. The diagnosis was suspected to be a space-occupying lesion in the larynx. On physical examination, the neonate had a clear mind, normal reflexes, pink cheeks, and a swelling in the left neck with a soft palpable 5 × 5-cm mass with an unclear boundary. His skin temperature and respiration rate was normal. Auscultation of both lungs revealed coarse breath sounds without rales and normal heart sounds.

**Figure 2:**
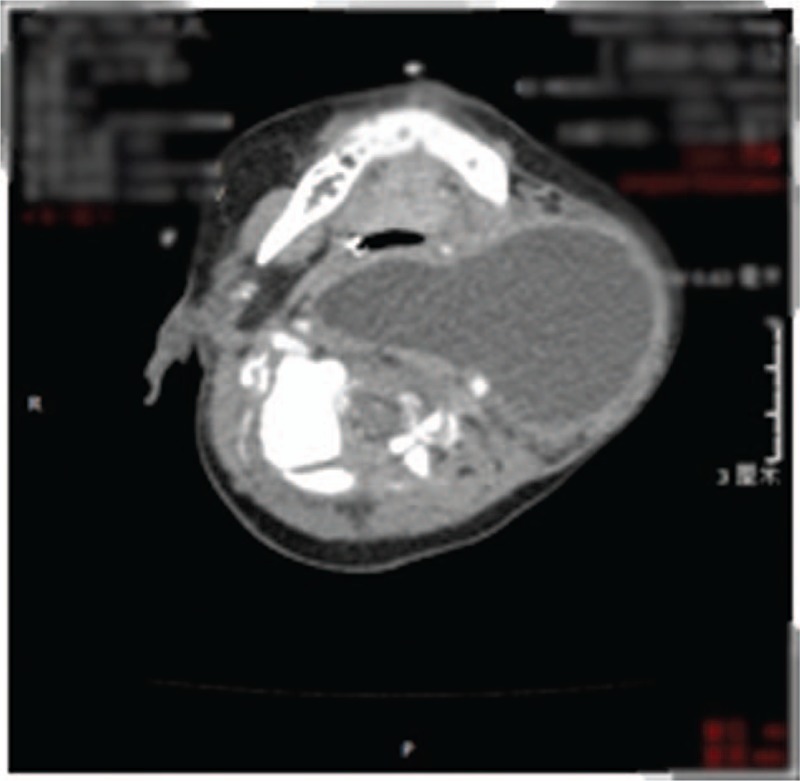
Axial contrast-enhanced CT scan image showing an abscess cavity with an irregularly enhanced cystic wall.

A chest X-ray suggested an increase in lung markings on both sides. No abnormality was found on color Doppler ultrasonography of the head. Echocardiography revealed a left-to-right (horizontal) shunt, an atrial septal defect, and a patent ductus arteriosus. A B-mode ultrasonographic examination of the left side of the neck revealed an anterior cervical cystic mass of indeterminate nature. Routine blood, urine, stool, electrolyte, and liver and kidney function tests were unremarkable and myocardial enzyme levels were normal. Blood culture showed no bacterial growth. A B-mode ultrasonogram obtained at 39 weeks’ gestation had revealed a 2.8 × 2.6-cm anechoic mass in the left side of the neck with the esophagus and trachea shifted to the right. A further B-mode ultrasonogram obtained one day after birth revealed a 5.1 × 3.8 × 3.1-cm cystic mass extending from the posterior pharynx and left side of the trachea to the front of the ascending aorta beneath, with no obvious displacement of the trachea but forward and outward movement of the jugular vein. The mass was observed to be regular in shape with a visible capsule and a clear boundary. No obvious blood flow signals were found in the mass on color Doppler flow imaging. On the 6th day after birth, B-mode ultrasonography showed a 6.6 × 5.0 × 3.9-cm cystic mass in the deep cervical fascial space on the left side; the mass had a clear boundary, an irregular shape, and a thick cystic wall, with dense spot-like echoes and gas-like echoes in the cystic cavity but no substantial space-occupying echoes. The upper lateral margin of the mass was adjacent to the left submandibular and parotid glands, and the lower margin extended to the thymus gland; it was adjacent to the left carotid sheath on the deep side with no wrapping and adjacent to the esophagus and left thyroid lobe on the medial side. A portion of the mass extended through the posterior part of the trachea to the inside of the right carotid sheath. Color Doppler flow imaging revealed spot-like blood flow signals in the wall of the cyst but no obvious blood flow signals in the mass. Axial contrast-enhanced CT scan image showing an abscess cavity with an irregularly enhanced cystic wall (Fig. 2).

**Figure 3:**
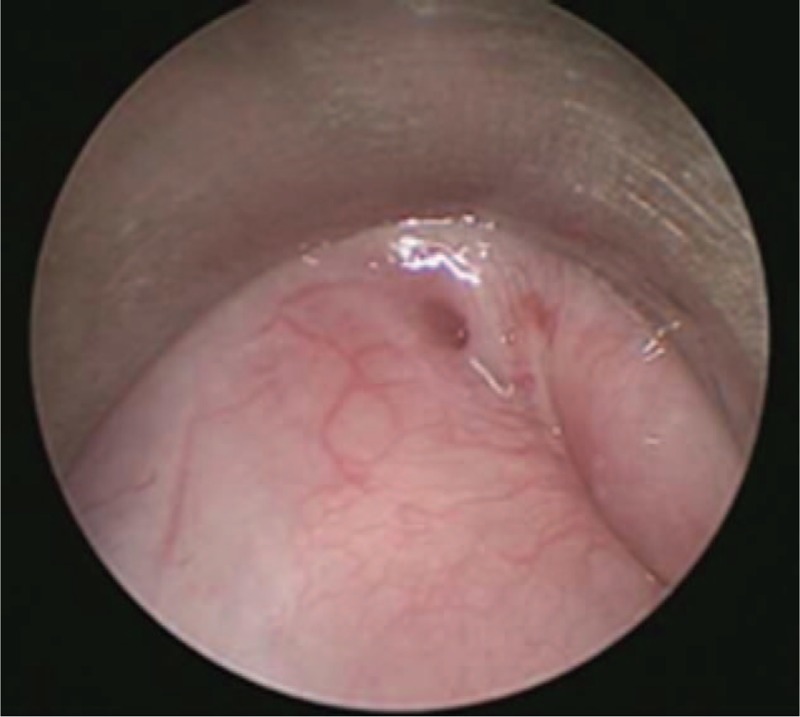
Suspension laryngoscopic image displaying the internal orifice in the recess of the left pyriform fossa.

**Figure 4:**
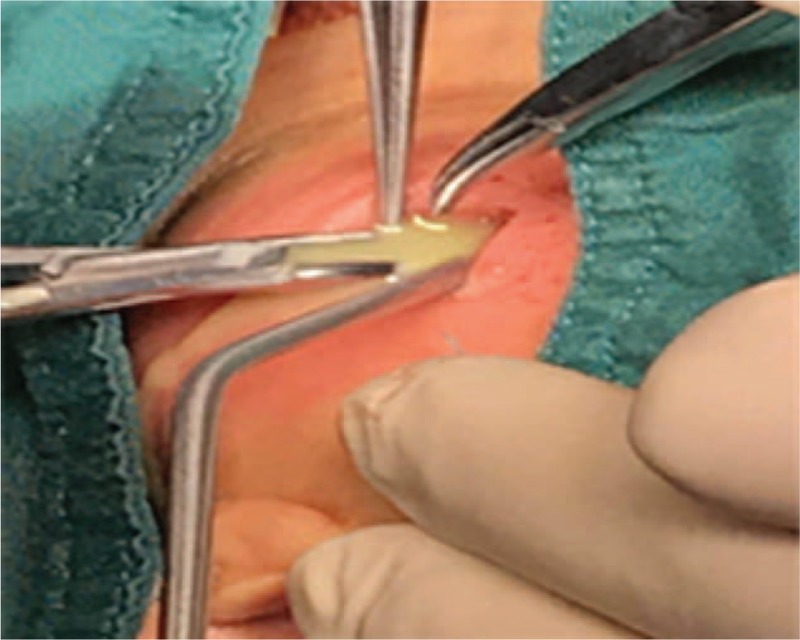
Incision and drainage of neck abscess intraoperatively.

**Figure 5:**
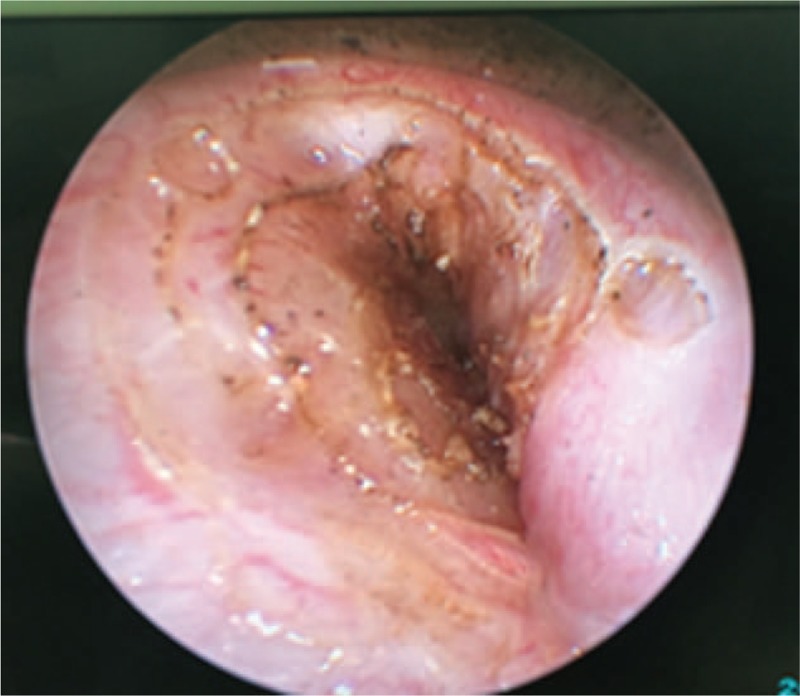
Endoscopic CO2 laser cauterization of the left internal orifice intraoperatively.

The neck abscess was incised and drained under general anesthesia. Approximately 200 ml of foul-smelling pus was drained intraoperatively (Fig. 3). Examination under suspension laryngoscopy indicated a pyriform sinus fistula on the left side (Fig. 4). Laser cauterization was performed simultaneously. Under the surgical microscope, the fistula and surrounding mucosa of the pyriform sinus were burned using a CO_2_ laser at a frequency of 2 W in continuous output mode. An area of basal mucosa in the pyriform sinus with a radius of 10 mm was burned, taking the internal fistula as the center (Fig. 5).

**Figure 6:**
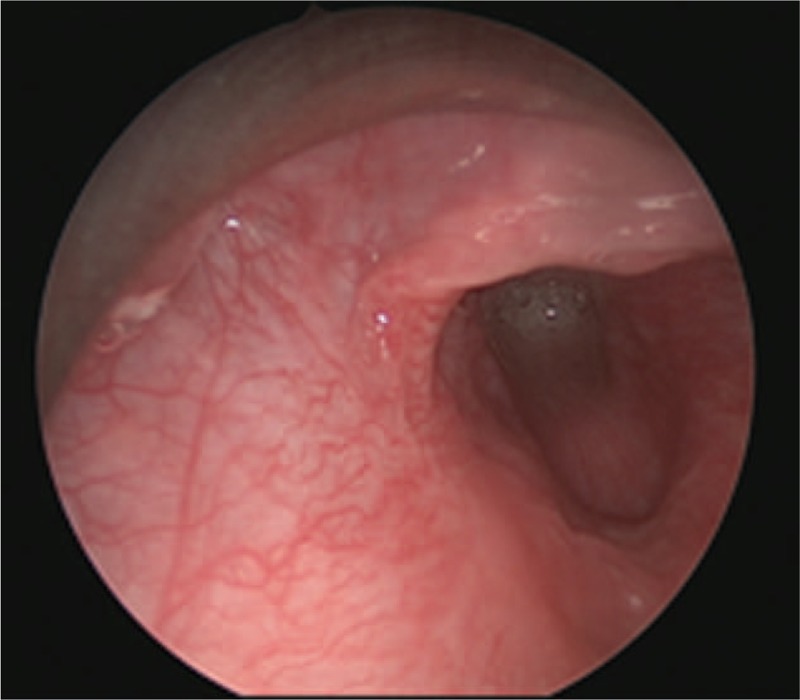
Three months after the endoscopic cauterization, suspension laryngoscopy showed that the left internal orifice was completely closed.

After surgery, the wound was dressed and anti-inflammatory treatment was provided. The neck wound healed uneventfully thereafter. Three months later, the fistula was confirmed to be closed by suspension laryngoscopy under general anesthesia (Fig. 6). No recurrence was detected during 9 months of follow-up.

## 3 Discussion

CPSF is a branchial abnormality originating from the third or fourth branchial pouch and is an important cause of anterior cervical abscess in children. The lesion mainly occurs on the left side and may be associated with asymmetric development of the original great artery and bilateral branchial apparatus or a C-cell migration disorder.^[6,7]^ A CPSF can be clinically classified as a sinus, that is, a blind-ending tract that opens to either the pyriform fossa or the skin, a cyst, which is not open to either, or a fistula, which is a tract connecting the pyriform fossa and skin. A sinus CPSF is most commonly encountered, although a fistula may develop after iatrogenic or spontaneous rupture of a cervical abscess. At least 80% of pyriform sinus fistulae occur in preschool children and the incidence is similar in both sexes.^[8]^ There is a general lack of awareness of this disease among non-specialist clinicians, which often leads to misdiagnosis and inappropriate treatment.

Patients with a pyriform sinus fistula usually show recurrent cervical swelling, pain, and an external opening with purulent discharge at the anterior border of the middle or lower third of the sternocleidomastoid muscle after an upper respiratory tract infection, often with symptoms of acute suppurative thyroiditis.^[9]^ However, because of the abundant blood supply and thick fibrous capsule in thyroid tissue, these lesions rarely become infected under normal conditions.^[10]^ Accordingly, the possibility of this disease should be considered in a patient with an acute suppurative thyroid inflammatory response, especially if it is on the left side and recurrent. Prompt investigation is needed to make a definitive diagnosis and to exclude other causes of a neck mass, such as a thyroglossal cyst or a second branchial cleft.

The diagnosis relies not only on clinical presentation but also on radiological findings. A barium swallow X-ray can reveal a sinus tract in the pyriform fossa, but the sensitivity of this method is only 50% to 80%.^[11,12]^ Both MRI and CT can identify a tract between the internal orifice and the neck mass or infection. MRI can detect soft tissue inflammation in the affected area.^[12]^ CT is considered to be more effective and requires a shorter scanning time than MRI.^[12,13]^ CT typically identifies inflammation and/or an abscess near the lesser horn of the thyroid cartilage, with air bubbles forming in the lesion or along the putative tract or may reveal a loss of high density in the thyroid gland.^[14]^ Further confirmation of an internal orifice in the pyriform fossa by suspension laryngoscopy or esophagoscopy is the gold standard for diagnosis of CPSF.

The external component of a pyriform sinus fistula is often absent and the internal fistula is concealed, which increases the likelihood of delayed diagnosis and treatment and leads to a relatively high rate of missed diagnosis and inappropriate treatment. Suspicion for CPSF should be high in a patient with an unexplained neck abscess or recurrent acute suppurative thyroiditis, especially if it occurs on the left side. Surgical treatment, such as endoscopic cauterization or open surgery, is required as soon as the diagnosis is made.^[15]^

A multicenter, prospective, randomized controlled study that includes long-term clinical follow-up is needed to investigate the advantages of surgical methods. Improvement in our understanding of this condition should lead to more effective methods of diagnosis and treatment. In summary, CPSF should be considered in neonates with a congenital cystic mass in the neck and in older pediatric patients with this presentation, even if there is no history of recurrent neck infection. In this case, a congenital cystic mass in the neck was found in the fetus and diagnosed as CPSF after birth, as opposed to repeated neck infections previously reported.

## 4 Consent for publication

The need for approval to publish this report was waived by the Institutional Review Board of Shenzhen Children's Hospital. However, written informed consent was obtained from the patient's parents for publication of the details of the patient's case and the accompanying images.

## Author contributions

**Data curation:** Saihong Han.

**Investigation:** Shuling Huang.

**Project administration:** Yishu Teng.

**Resources:** Shuling Huang.

**Supervision:** Shuling Huang, Zhixiong Xian.

**Visualization:** Guowei Chen, Lan Li.

**Writing – original draft:** Yishu Teng.

**Writing – review & editing:** Yishu Teng.

## References

[1] Liu T Wen Z Liang QF The diagnosis and treatment for pyriform sinus fistula in children. Nat Med China 2016;96:3156–9. 10.3760/cma.j.issn.0376-2491.2016.39.009 27852415

[2] Ford GR Balakrishnan A Evans JN Branchial cleft and pouch anomalies. Laryngol Otol 1992;106:137–43. 10.1017/s0022215100118900 1556487

[3] Zhang P Tian X Recurrent neck lesions secondary topyriform sinus fistula. Eur Arch Otorhinolaryngol 2016;273:735–9. 25708412 10.1007/s00405-015-3572-2

[4] Parida PK Gopalakrishnan S Saxena SK Pediatric recurrent acute suppurative thyroiditis of third branchial arch origin-our experience in 17 cases. Int J Pediatr Otorhinolaryngol 2014;78:1953–7. 25219934 10.1016/j.ijporl.2014.08.034

[5] Masuoka H Miyauchi A Tomoda C Imaging studies in sixty patients with acute suppurative thyroiditis. Thyroid 2011;21:1075–80. 21875365 10.1089/thy.2010.0366

[6] Nicoucar K Giger R Jaecklin T Management of congenital third branchial arch anomalies: a systematic review. Otolaryngol Head Neck Surg 2010;142:21–8. e2. 20096218 10.1016/j.otohns.2009.09.001

[7] Nicoucar K Giger R Pope HG Jr Management of congenital fourth branchial arch anomalies: a review and analysis of published cases. J Pediatr Surg 2009;44:1432–9. 19573674 10.1016/j.jpedsurg.2008.12.001

[8] Thomas B Shroff M Forte V Revisiting imaging features and the embryologic basis of third and fourth branchial anomalies. AJNR Am J Neuroradiol 2010;31:755–60. 20007720 10.3174/ajnr.A1902 PMC7964224

[9] Lachance S Chadha NK Systematic review of endoscopic obliteration techniques for managing congenital piriform fossa sinus tracts in children. Otolaryngol Head Neck Surg 2015;154:241–6. 26527612 10.1177/0194599815613286

[10] Lyu P Feng B Diagnosis and treatment of congenital piriform fistula. Int Otolaryngolayg Head Surg 2012;36:157–8.

[11] Yolmo D Madana J Kalaiarasi R Retrospective case review of pyriform sinus fistulae of third branchial arch origin commonly presenting as acute suppurative thyroiditis in children. J Laryngol Otol 2012;126:737–42. doi: 10.1017/S0022215112000898. 22624855 10.1017/S0022215112000898

[12] Watson GJ Nichani JR Rothera MP Case series: endoscopic management of fourth branchial arch anomalies. Int J Pediatr Otorhinolaryngol 2013;77:766–9. doi: 10.1016/j.ijporl.2013.02.007. 23478017 10.1016/j.ijporl.2013.02.007

[13] Liang Lu Chen Liangsi Zhou Zhenggen Comparative imaging studies of congenital pyriform sinus fistula [in Chinese]. Chin J Radiol 2016;50:196–200.

[14] Wang HK Tiu CM Chou YH Imaging studies of pyriform sinus fistula. Pediatr Radiol 2003;33:328–33. doi: 10.1007/s00247-003-0887-8. 12695866 10.1007/s00247-003-0887-8

[15] Derks LSM Veenstra HJ Oomen KPQ Surgery versus endoscopic cauterization in patients with third or fourth branchial pouch sinuses: a systematic review. Laryngoscope 2016;126:212–7. 26372400 10.1002/lary.25321

